# Perceived Discrimination Among Food Pantry Clients in Massachusetts

**DOI:** 10.5888/pcd21.240009

**Published:** 2024-09-12

**Authors:** Cara F. Ruggiero, Man Luo, Rachel M. Zack, James P. Marriott, Catherine Lynn, Daniel Taitelbaum, Paige Palley, Aprylle M. Wallace, Norbert Wilson, Angela Odoms-Young, Lauren Fiechtner

**Affiliations:** 1 Division of General Academic Pediatrics, Department of Pediatrics, Mass General for Children, Boston, Massachusetts; 2 Now with University of Cambridge School of Clinical Medicine, Cambridge, United Kingdom; 3 Business and Data Analytics Department, The Greater Boston Food Bank, Boston, Massachusetts; 4 Communcation and Public Affairs, The Greater Boston Food Bank, Boston, Massachusetts; 5 Human Resources and Diversity, Equity, and Inclusion, The Greater Boston Food Bank, Boston, Massachusetts; 6 Duke Divinity School, Sanford School of Public Policy, Duke University, Durham, North Carolina; 7 Division of Nutritional Sciences, College of Human Ecology, Cornell University, Ithaca, New York; 8 Division of Pediatric Gastroenterology and Nutrition, Mass General for Children, Boston, Massachusetts

## Abstract

**Introduction:**

Food insecurity is defined as inconsistent access to enough food to meet nutritional needs. Discrimination is associated with food insecurity and poor health, especially among racial and ethnic minoritized and sexual or gender minoritized groups. We examined the demographic associations of perceived everyday discrimination and food pantry discrimination in Massachusetts.

**Methods:**

From December 2021 through February 2022, The Greater Boston Food Bank conducted a cross-sectional, statewide survey of Massachusetts adults. Of the 3,085 respondents, 702 were food pantry clients for whom complete data on food security were available; we analyzed data from this subset of respondents. We used the validated 10-item Everyday Discrimination Scale to measure perceived everyday discrimination and a 10-item modified version of the Everyday Discrimination Scale to measure perceived discrimination at food pantries. Logistic regression adjusted for race and ethnicity, age, gender identity, sexual orientation, having children in the household, annual household income, and household size assessed demographic associations of perceived everyday discrimination and discrimination at food pantries.

**Results:**

Food pantry clients identifying as LGBTQ+ were more likely than those identifying as non-LGBTQ+ to report perceived everyday discrimination (adjusted odds ratio [AOR] = 2.44; 95% CI, 1.24–4.79). Clients identifying as Hispanic (AOR = 1.83, 95% CI, 1.13–2.96) were more likely than clients identifying as non-Hispanic White to report perceived discrimination at food pantries.

**Conclusion:**

To equitably reach and serve households with food insecurity, food banks and pantries need to understand experiences of discrimination and unconscious bias to develop programs, policies, and practices to address discrimination and create more inclusive interventions for food assistance.

**Summary**

**What is already known on this topic?**

Disparities in rates of food insecurity exist among socially marginalized groups in Massachusetts and nationwide. Perceived discrimination has been linked to food insecurity, physical health problems, and risk factors for diseases such as obesity, high blood pressure, depression, and substance use.

**What is added by this report?**

Our analysis provides preliminary evidence of perceived discrimination related to age, income, gender identity, and race at food pantries in a diverse sample of food pantry clients in Massachusetts.

**What are the implications for public health practice?**

Public health interventions and food assistance programs focused on improving food security may not reach maximum effectiveness without addressing discrimination.

## Introduction

Food insecurity is defined as inconsistent access to sufficient food to meet nutritional needs (1). Food insecurity exists on a continuum, starting with anxiety and progressing to reduced food intake. High food security is defined as no indication of food access challenges. Marginal food security is defined as 1 or 2 indications of food access challenges (eg, anxiety over food sufficiency or shortage of food in the house). Low food security is defined as reports of reduced quality, variety, or desirability of diet with little or no indication of changes in diets or food intake. Very low food security is defined as reports of multiple indications of disrupted eating patterns and reduced food intake (2). Adverse health outcomes associated with food insecurity are well evidenced, such as greater odds of hospitalization and fair or poor health among infants and toddlers (3), higher odds of obesity among children (4,5), and higher rates of type 2 diabetes among adults (6). These problems may have been exacerbated by the COVID-19 pandemic (7).

Although studies indicate that contributors to food insecurity are multifactorial, key drivers are systemic inequities in society, including structural racism, generational poverty, unconscious bias, homophobia, and transphobia across many sectors (8–11). Racial, ethnic, and socioeconomic disparities in food insecurity are long-standing, with studies indicating that groups who are socially marginalized experience higher rates of food insecurity and greater severity (ie, low or very low food security) (12) than groups that are not socially marginalized. For example, households headed by Black and Hispanic individuals, households living at or below the poverty level, and households headed by a single adult have a higher prevalence of food insecurity compared with the US population overall (13). Many disparities in food insecurity have persisted since the federal government first started measuring food insecurity 20 years ago (8). At the intersection of systemic, individual, and household factors, perceived discrimination is associated with a higher risk of food insecurity (10,14–16). Perceived discrimination is defined as a person’s perception of negative attitude, judgment, or unfair treatment from others because of characteristics such as gender, race, ethnicity, and social status (17) and is linked to physical health problems and risk factors for disease, such as obesity, high blood pressure, depression, and substance use (18,19). Racism (perceived or not) is a source of acute and chronic stress for minoritized groups and may be an important mediator in the pathway from perceived discrimination to adverse health outcomes (20). Furthermore, the subjective nature of perceived discrimination prevents a person from being able to determine the stressfulness of a situation a priori (ie, perception of harm or threat) (21). Food insecurity and perceived discrimination are linked to health outcomes (18,19); however, the food banking system can help alleviate food insecurity (22). The food banking system (23) (ie, emergency food system or charitable food system) (24) is a robust network of food service models that includes food banks (organizations that source, store, and distribute food) and food pantries (community agencies where individuals pick up food at no or limited cost) (25). The Greater Boston Food Bank (GBFB) is the largest food bank in New England. This network includes 600 food pantries and serves 600,000 people per year in eastern Massachusetts (26). GBFB is a member organization of Feeding America, a network of more than 200 food banks serving 60,000 food pantries (26). GBFB aims to eliminate the root causes of food insecurity by working to promote racial, gender, and economic equity to achieve social justice. However, research on perceived discrimination experienced at food pantries is lacking and could be a barrier to minoritized populations accessing the charitable food system (27).

The objective of this secondary analysis was to measure experiences of perceived discrimination among food pantry clients in Massachusetts. We examined the associations of demographic characteristics with perceived everyday discrimination among food pantry clients and explored perceived discrimination experienced at Massachusetts food pantries.

## Methods

### Study design and participants

During the COVID-19 pandemic, from December 2021 through February 2022, GBFB and MassGeneral for Children administered a cross-sectional, statewide, representative survey in English of Massachusetts adults (N = 3,085 respondents). The Massachusetts Statewide Food Access Survey was developed by the National Food Access and COVID Research Team (28) and modified with input from the Health and Research Council at GBFB, which includes experts from the nonprofit, government, medical, legal, health equity, and public health sectors (26). Survey participants were recruited by Qualtrics, a survey research firm, to complete an online survey on food access and food security through the Qualtrics Panels Project. Qualtrics recruits participants meeting study inclusion criteria from many panel sources. Exact information on compensation for participants is unavailable because this information is kept confidential by Qualtrics. All recruitment, participant contact, data collection, and compensation were handled by Qualtrics directly. Qualtrics has numerous data quality checks in place (eg, removing duplicate internet protocol addresses) to ensure that only 1 survey was completed per household. GBFB and MassGeneral for Children received the anonymous data after it was collected by Qualtrics.

The objectives of the Massachusetts Statewide Food Access Survey were to identify changes in the prevalence of food insecurity and use of food assistance, document barriers to and facilitators of food pantry use and SNAP (Supplemental Nutrition Assistance Program) participation, and develop data-driven recommendations to improve food access equity through programmatic, policy, and advocacy efforts (29). Of 5,064 entrants to the survey, 4,336 participants completed the survey, and 3,085 completed surveys were of good quality. This secondary analysis was restricted to an unweighted sample of food pantry clients with complete data on food insecurity (n = 702). This study was approved by the institutional review board at MassGeneral for Children.

### Measures

Demographic questions asked survey participants to self-identify their race and ethnicity, age category, sexual orientation (30), and gender identity (30). Race and ethnicity were defined according to US Census Bureau methods (31). Hispanic ethnicity was defined as any adult identifying as Hispanic, regardless of identity with a racial group (eg, Black Hispanic was defined as Hispanic). Additionally, when possible, data were further disaggregated such that free-text responses for “other” were disaggregated and categorized into the appropriate category (eg, Brazilian or Puerto Rican was counted as Hispanic) to attempt to highlight disparities in adults identifying as Hispanic. In sensitivity analyses, we then used an aggregated variable for race and ethnicity that was consistent with the US Census Bureau method. For both variables, if a participant chose more than 2 races and did not identify as Hispanic or Latino, they were categorized as “other.” Participants were also asked to report the number of children in the household, annual household income, and household size (number of household members).

The survey asked about food insecurity and use of food assistance programs. Food insecurity during the last year was assessed by using the US Department of Agriculture’s 6-item short form of the food security survey module (32), and food insecurity was defined as having at least 2 affirmative responses (eg, often, sometimes, or yes). The 6-item measure was chosen because of its focus on adults and the study team’s desire to reduce the length of the survey. SNAP participation was assessed by using a single item that asked participants whether they had received SNAP benefits in the last year, the last 30 days, never, or used more than a year ago, but not currently using. For this analysis, respondents were considered SNAP participants if they had reported using the program in the last year or the last 30 days.

Perceived everyday discrimination was measured by using the validated 10-item Everyday Discrimination Scale developed by Williams and colleagues (20). The questionnaire first asks the question, “In your day-to-day life, how often do any of the following things happen to you?” Nine statements follow, such as “You are treated with less courtesy than other people are.” The 6 response options were almost every day, at least once a week, a few times a month, a few times a year, less than once a year, and never. Any respondent who reported almost every day, at least once a week, a few times a month, or a few times a year to any of the 9 statements was categorized as experiencing perceived everyday discrimination (19). These participants were then asked a follow-up question: “What do you think is the main reason for these experiences?” Participants could select multiple reasons from a list of 10 reasons: ancestry or national origins, gender, race, age, religion, height, weight, some other aspect of your physical appearance, sexual orientation, education or income level.

Perceived discrimination at food pantries was explored by using a modified version of the Everyday Discrimination Scale (Box). The first question was, “At a food pantry, how often do any of the following things happen to you?” The 9 statements were identical to those in the original questionnaire. Responses included all of the time, most of the time, some of the time, rarely, and never. Any respondent who reported all of the time, most of the time, or some of the time to any of the 9 questions was categorized as experiencing perceived discrimination at food pantries. Again, respondents were asked to select what they thought was the main reason for their experiences.

**Box. Questions on Perceived Food Pantry Discrimination in a Survey Administered by the Greater Boston Food Bank and Mass General for Children, December 2021–February 2022^a^**

**At a food pantry, how often do any of the following things happen to you? Response options for all questions were all of the time, most of the time, some of the time, rarely, never.**

□ You are treated with less courtesy than other people are

□ You are treated with less respect than other people are

□ You receive poorer service than other people

□ People act as if they think you are not smart

□ People act as if they are afraid of you

□ People act as if they think you are dishonest

□ People act as if they’re better than you are

□ You are called names or insulted

□ You are threatened or harassed

**What do you think is the main reason for these experiences? (Check all that apply)**

□ My ancestry or national origins

□ My gender

□ My race

□ My age

□ My religion

□ My height

□ My weight

□ Some other aspect of my physical appearance

□ My sexual orientation

□ My education or income level

□ My mental health condition

□ My physical disability

^a^ Modified from the Everyday Discrimination Scale developed by Williams and colleagues (20).

### Data analysis

We examined descriptive characteristics and bivariate associations among the exposures age, race and ethnicity, gender identity, sexual orientation, annual household income, household size, having children in the household, food insecurity, and SNAP participation. Then, logistic regression examined the adjusted associations of the exposures with perceived everyday discrimination and perceived discrimination at food pantries. Fully adjusted models included age, race, gender identity, sexual orientation, annual household income, household size, having children in the household, food insecurity, and SNAP participation. We used SAS version 9.4 (SAS Institute, Inc) for all analyses. The level of significance was set at *P* <.05.

## Results

Most food pantry clients in our sample identified as non-Hispanic White (59%) and cisgender women (64%); 22% identified as Hispanic and 11% as non-Hispanic Black. Additionally, 40% were aged 18 to 34 years, 44% had children in the household, 23% identified as LGBTQ+, and 42% had an annual household income of less than $25,000 (Table 1).

**Table 1:** Demographic Characteristics of Food Pantry Clients (N = 702) Who Responded to a Survey Administered by the Greater Boston Food Bank and Mass General for Children, December 2021–February 2022

Characteristic	No. (%)^a^
**Race and ethnicity**	
Hispanic^b^	153 (22)
Non-Hispanic Asian	32 (5)
Non-Hispanic Black	77 (11)
Non-Hispanic White	417 (59)
Non-Hispanic Other	23 (3)
**Age, y**	
18–34	284 (40)
35–54	273 (39)
55–64	90 (13)
≥65	55 (8)
**Gender identity**	
Cisgender man	237 (34)
Cisgender woman	447 (64)
Nonbinary/transgender	18 (3)
**Sexual orientation**	
Non-LGBTQ+	541 (77)
LGBTQ+	161 (23)
**Have children in household**	306 (44)
**Annual household income, $**	
<25,000	298 (42)
25,000–49,999	202 (29)
50,000–74,999	103 (15)
75,000–99,999	41 (6)
≥100,000	58 (8)
**Region of Massachusetts**	
Boston	100 (14)
Central	98 (14)
Northeast	177 (25)
Southeast	182 (26)
Western	145 (21)

Abbreviation: LGBTQ, lesbian, gay, bisexual, transgender, queer.

a Percentages may not add to 100 because of rounding.

b Defined as any adult identifying as Hispanic, regardless of identity with a racial group (eg, Black Hispanic was defined as Hispanic).

### Demographic associations with perceived everyday discrimination

In adjusted models examining the association of demographic characteristics with perceived everyday discrimination (Table 2), clients aged 18 to 34 years were more likely than clients aged 65 years or older to report perceived everyday discrimination (adjusted odds ratio [AOR] = 4.27; 95% CI, 1.89–9.67). Clients identifying as LGBTQ+ were more likely than non-LGBTQ+ clients to report perceived everyday discrimination (AOR = 2.44; 95% CI, 1.24–4.79). Clients with annual household incomes of $100,000 or more were more likely than clients with incomes of less than $25,000 annually to report perceived everyday discrimination (AOR = 4.51; 95% CI, 1.52–13.31). Clients experiencing food insecurity (AOR = 4.49; 95% CI, 2.79–7.22) were more likely than clients not experiencing food insecurity to report perceived everyday discrimination. We found no differences in perceived everyday discrimination by race.

**Table 2:** Adjusted Logistic Regression of Perceived Everyday Discrimination and Food Pantry Discrimination Among Food Pantry Clients (N = 702) Who Responded to a Survey Administered by the Greater Boston Food Bank and Mass General for Children, December 2021–February 2022

Characteristic	Perceived everyday discrimination		Perceived food pantry discrimination	
	Reporting yes, %	Adjusted OR (95% CI)	Reporting yes, %	Adjusted OR (95% CI)
**Race and ethnicity^a^**				
Hispanic	89	1.16 (0.60–2.23)	69	1.83 (1.13–2.96)
Non-Hispanic Asian	84	1.70 (0.51–5.61)	53	1.11 (0.46–2.65)
Non-Hispanic Black	87	1.31 (0.59–2.94)	61	1.63 (0.89–2.95)
Non-Hispanic White	79	1 [Reference]	47	1 [Reference]
Non-Hispanic Other	87	1.06 (0.28–4.02)	61	1.49 (0.54–4.16)
**Age, y**				
18–34	90	4.27 (1.89–9.67)^b^	69	1.73 (0.80–3.73)
35–54	84	2.09 (1.00–4.35)	51	0.89 (0.42–1.88)
55–64	67	1.07 (0.50–2.29)	33	0.83 (0.36–1.91)
≥65	62	1 [Reference]	33	1 [Reference]
**Gender identity^c^**				
Man	86	1 [Reference]	51	1 [Reference]
Woman	81	0.79 (0.48–1.28)	60	0.76 (0.52–1.12)
**Sexual orientation**				
Non-LGBTQ+	80	1 [Reference]	51	1 [Reference]
LGBTQ+	92	2.44 (1.24–4.79)^b^	66	1.51 (0.98–2.33)
**Have children in household**				
No	80	1 [Reference]	48	1 [Reference]
Yes	87	0.92 (0.52–1.62)	62	1.19 (0.77–1.84)
**Annual household income, $**				
<25,000	80	1 [Reference]	54	1 [Reference]
25,000–49,999	84	1.59 (0.93–2.74)	53	0.94 (0.61–1.43)
50,000–74,999	83	1.45 (0.73–2.88)	47	0.59 (0.34–1.01)
75,000–99,999	80	1.61 (0.61–4.22)	46	0.73 (0.33–1.63)
≥100,000	91	4.51 (1.52–13.31)^b^	78	3.53 (1.55–8.03)^b^
**Household size, mean (SD)**	2.8 (1.5)	0.91 (0.76–1.09)	2.8 (1.5)	1.03 (0.89–1.19)
**SNAP participation**				
No	75	1 [Reference]	47	1 [Reference]
Yes	85	1.56 (0.94–2.61)	57	0.98 (0.63–1.51)
**Experiencing food insecurity**				
No	60	1 [Reference]	26	1 [Reference]
Yes	88	4.49 (2.79–7.22)^b^	61	3.25 (1.97–5.37)^b^
**Perceived everyday discrimination**				
No	**-**	1 [Reference]	9	1 [Reference]
Yes	**-**	**-**	64	11.20 (5.72–21.90)^b^

Abbreviations: LGBTQ, lesbian, gay, bisexual, transgender, queer; OR, odds ratio; SNAP, Supplemental Nutrition Assistance Program.

a In this primary analysis, 32 respondents were non-Hispanic Asian, 77 non-Hispanic Black, 153 Hispanic, 417 non-Hispanic White, and 23 non-Hispanic “Other.” Hispanic was defined as any adult identifying as Hispanic, regardless of identity with a racial group (eg, Black Hispanic was defined as Hispanic). In addition, when possible, data were disaggregated such that free-text responses for “Other” were disaggregated and categorized into the appropriate category (eg, Brazilian or Puerto Rican was counted as Hispanic).

b Significant at *P* < .05.

c 2-level sex category was used because cell size of nonbinary/transgender was too small and model did not converge. These individuals were instead captured in the LGBTQ+ variable.

### Demographic associations with perceived discrimination at food pantries

In adjusted models examining the association of demographic characteristics with perceived discrimination at food pantries (Table 2), clients identifying as Hispanic were more likely than clients identifying as non-Hispanic White to report perceived discrimination at food pantries (AOR = 1.83; 95% CI, 1.13–2.96). However, when we used the aggregated race and ethnicity variable, we found that clients identifying as Black were more likely than clients identifying as White to report perceived discrimination at food pantries (AOR = 1.91; 95% CI, 1.02–3.60) (Table 3). Clients with annual household incomes of $100,000 or more were more likely than clients with incomes of less than $25,000 annually to report perceived discrimination at food pantries (AOR = 3.53; 95% CI, 1.55–8.03) (Table 2). Clients reporting everyday discrimination were more likely than clients not reporting everyday discrimination to report perceived discrimination at food pantries (AOR = 11.34; 95% CI, 5.79–22.24) (Table 3). Of the 702 food pantry clients, 580 (83%) reported everyday discrimination and 381 (54%) reported discrimination at food pantries. The most common reasons reported were related to weight, age, race, and gender identity (Table 4).

**Table 3:** Adjusted Logistic Regression on Perceived Everyday Discrimination and Food Pantry Discrimination, by Aggregated Race Variable, Among Food Pantry Clients (N = 702) Who Responded to a Survey Administered by the Greater Boston Food Bank and Mass General for Children, December 2021–February 2022^a^

Characteristic	Odds ratio (95% CI)	
	Perceived everyday discrimination	Perceived food pantry discrimination
**Race**		
Asian	1.36 (0.40–4.61)	0.74 (0.30–1.88)
Black	1.14 (0.50–2.56)	1.91 (1.02–3.60)^b^
Hispanic	1.15 (0.60–2.21)	1.84 (1.14–2.97)^b^
White	1 [Reference]	1 [Reference]
Other	1.96 (0.54–7.10)	1.46 (0.63–3.37)
**Age, y**		
18–34	4.35 (1.92–9.88)^b^	1.75 (0.81–3.80)
35–54	2.05 (0.98–4.27)	0.90 (0.42–1.91)
55–64	1.05 (0.49–2.25)	0.84 (0.36–1.92)
≥65	1 [Reference]	1 [Reference]
**Gender identity** c		
Man	1 [Reference]	1 [Reference]
Woman	0.79 (0.49–1.29)	0.75 (0.51–1.11)
**Sexual orientation**		
Non-LGBTQ+	1 [Reference]	1 [Reference]
LGBTQ+	2.37 (1.21–4.64)^b^	1.48 (0.96–2.29)
**Have children in household**		
No	1 [Reference]	1 [Reference]
Yes	0.91 (0.52–1.61)	1.18 (0.77–1.82)
**Annual household income, $**		
<25,000	1 [Reference]	1 [Reference]
25,000–49,999	1.58 (0.92–2.72)	0.93 (0.61–1.42)
50,000–74,999	1.46 (0.73–2.91)	0.57 (0.33–0.99)^b^
75,000–99,999	1.62 (0.62–4.25)	0.72 (0.32–1.61)
≥100,000	4.69 (1.58–13.90)^b^	3.65 (1.59–8.35)^b^
**Household size, mean (SD)**	0.92 (0.77–1.09)	1.04 (0.89–1.20)
**SNAP participation**		
No	1 [Reference]	1 [Reference]
Yes	1.56 (0.93–2.59)	0.97 (0.63–1.51)
**Experiencing food insecurity**		
No	1 [Reference]	1 [Reference]
Yes	4.47 (2.77–7.19)^b^	3.23 (1.96–5.34)^b^
**Perceived everyday discrimination**		
Yes	—	11.34 (5.79–22.24)^b^
No	—	1 [Reference]

Abbreviations: —, does not apply; LGBTQ, lesbian, gay, bisexual, transgender, queer; SNAP, Supplemental Nutrition Assistance Program.

a In this analysis, which used an aggregated race and ethnicity variable, 28 respondents were Asian, 71 Black, 153 Hispanic, 417 White, and 33 “Other.”

b *P* < .05.

c 2-level sex category used because cell size for nonbinary/transgender was too small and model did not converge. Data for nonbinary/transgender were instead captured in the LGBTQ+ variable.

**Table 4:** Prevalence of Discrimination and Frequencies of Reasons for Discrimination Reported by Food Pantry Clients (N = 702) Who Responded to a Survey Administered by the Greater Boston Food Bank and Mass General for Children, December 2021–February 2022

Characteristic	Everyday discrimination, no. (%)	Discrimination at food pantries, no. (%)
**Experienced discrimination in the last year and reported a reason**	580 (83)	381 (54)
**Reasons reported^a^**		
Weight	173 (30)	120 (31)
Age	169 (29)	128 (34)
Race	169 (29)	118 (31)
Gender identity	170 (29)	100 (26)
Mental health condition	162 (28)	84 (22)
Other physical discrimination	139 (24)	95 (25)
Socioeconomic status	138 (24)	76 (20)
Disability	81 (14)	55 (14)
Ancestry	68 (12)	55 (14)
Height	70 (12)	61 (16)
Sexual orientation	71 (12)	51 (13)
Religion	63 (11)	59 (15)

a More than 1 response was permitted. Percentages are based on the number of pantry clients who reported discrimination and gave a reason (n = 580 for everyday discrimination and n = 381 for discrimination at food pantries).

## Discussion

Our analysis supports the link between perceived everyday discrimination and food insecurity and provides preliminary evidence on perceived discrimination at food pantries in a diverse sample of food pantry clients in Massachusetts. Our results suggest that clients aged 18 to 34 years (vs aged ≥65 y) and clients in households in the highest income category (vs the lowest) were more likely to report perceived everyday discrimination and discrimination at food pantries. Clients identifying as LGBTQ+ (vs non-LGBTQ+) were more likely to report everyday discrimination. Furthermore, clients identifying as Hispanic were more likely than clients identifying as non-Hispanic White to report perceived discrimination at food pantries.

Our findings are aligned with other findings on gender identity and sexual orientation (33,34) and racial and ethnic (35) disparities in food insecurity rates. Our findings are also aligned with other reports of higher rates of everyday discrimination among sexual and gender minoritized groups and racial and ethnic minoritized groups (36–38). However, our findings add to this literature by focusing on perceived discrimination among food pantry clients.

Our findings on food pantry clients who identified as Hispanic are noteworthy. While data on perceived discrimination at food pantries is scant, the Hispanic population in Massachusetts has a disproportionate share of health disparities, such as worse outcomes during the COVID-19 pandemic (39). The Hispanic population also experienced a disproportionate share of the effects of the pandemic, such as having inadequate sick leave or needing to reduce work hours to care for children (40). These factors could have contributed to greater perceived discrimination at food pantries and may offer one explanation as to why our findings on Hispanic food pantry clients were robust even after adjusting for covariates. Additionally, 51% of clients identifying as Hispanic in this sample noted that they were worried about documents they needed to provide at food pantries, even though documentation is not required in Massachusetts. This concern may contribute to greater levels of perceived discrimination at food pantries.

Our findings are also in agreement with other literature that documented discrimination in shopping and seeking food assistance. For example, in a qualitative study among transgender and gender-nonconforming individuals, participants feared gender-based discrimination from religious groups who organize food pantries, which kept them from seeking food assistance in local communities (41). Another qualitative study of young adults with diverse racial and ethnic backgrounds reported experiencing several forms of discrimination while shopping in food retail stores, such as excessive monitoring and verbal harassment tied to race and ethnicity and xenophobia, which influenced how their households acquired food (42).

Our finding on the association between higher income and perceived discrimination at food pantries was somewhat surprising. However, food pantry participants in households with higher incomes may have needed to use food pantries for the first time during COVID-19 pandemic, thus, they may have had a heightened sense of perceived discrimination, even though food pantry staff would not be aware of their income. Data from this sample indicate that that 11% of new pantry clients in the last year had annual incomes of $100,000 or more. This percentage is in line with national data from Feeding America, which indicated that the number of people participating in the charitable food system increased from 40 million in 2019, before the COVID-19 pandemic began, to 49 million in 2022, an increase of 22.5% (43). Thus, individuals who typically did not need food assistance before the pandemic were seeking assistance for the first time. Additionally, our findings about higher income and everyday discrimination are in line with other research showing that both income and education are directly and linearly associated with both presence of discrimination and intensity of discrimination (44).

Our results demonstrating that younger adults perceived more everyday discrimination than older adults were unexpected. While there is robust literature examining the experiences of discrimination among older adults, evidence is growing that other age groups also experience discrimination (45). Literature on discrimination among young adults apart from race and ethnicity is limited (46); however, a study that used data from 6 waves of the Transition to Adulthood Supplement (2007–2017) of the Panel Study of Income Dynamics found that more than 90% of young adults across racial and ethnic groups reported experiencing everyday discrimination (47). Younger age groups also may be more aware of discrimination and microaggressions, and thus be more likely to report it, but more work is needed to test this hypothesis.

It is important to acknowledge that findings about perceived discrimination at food pantries among clients identifying as Black were not significant when we used a disaggregated variable for race and ethnicity, which counted adults identifying as Hispanic and 1 other racial category as Hispanic (including Black Hispanic adults). This disaggregation method also assigned free-text responses in the “other” category to the closest appropriate category consistent with US Census Bureau methods (48). Challenges remain in the disaggregation of race data, and although no one-size-fits-all approach exists to collect, analyze, and report such data, it is important to prioritize transparency and ensure that all minoritized populations are represented and visible in research (49).

### Limitations and strengths

This study has several limitations and strengths. Because the statewide survey was available only in English, some barriers to and experiences with food pantries may have been underreported. Additionally, the survey was offered online only and, therefore, it was not available to individuals without access to computers or smartphones. However, data from 2021 showed that most Americans (93%) have internet access, even lower-income populations (50). The online nature of the survey also allowed participants to complete sensitive questions in the privacy of their own homes, potentially decreasing the possibility of social desirability bias. Finally, although the Everyday Discrimination Scale (32) was adapted with the help of content experts for use in food pantries, it has not undergone factor analysis. Future work will aim to conduct psychometric testing to establish reliability and validity. Strengths of the study include a diverse sample and collection of novel data on experiences of perceived discrimination among food pantry clients, thus adding to the existing literature on racial, ethnic, sexual, and gender disparities in food access.

### Conclusions

Results from this secondary analysis emphasize the urgent need to address discrimination, which may contribute to inequities in the charitable food system, particularly among racial, ethnic, gender identity, and sexual orientation minoritized groups. Public health interventions and food assistance programs aimed at improving food security may not reach maximum effectiveness without addressing discrimination. Integrating essential unconscious bias training is needed to protect racial and ethnic and gender identity and sexual orientation minoritized groups. Organizations should focus efforts on diversity, equity, and inclusion initiatives and work toward concrete solutions such as data disaggregation, which is both tangible and impactful. Researchers and practitioners at food banks and food pantries who are developing policies and programs should work with those with lived expertise to better understand the experiences and barriers caused by perceived discrimination to equitably serve all who could benefit from the charitable food assistance system.
